# What participants think about learning Mindfulness-Based Programs online

**DOI:** 10.1186/s40359-025-02975-8

**Published:** 2025-12-09

**Authors:** Alison M. Burton, Bridgette O’Neill, Gemma M. Griffith

**Affiliations:** Centre for Mindfulness Research and Practice, School of Psychology and Sports Science, Bangor University, Bangor, Wales UK

**Keywords:** Mindfulness, Mindfulness-Based Programs (MBPs), Online, Mental health, Telehealth, Pedagogy

## Abstract

**Objectives:**

Little is known about how participants experience Mindfulness-Based Programs (MBPs) that are delivered online. Understanding how online delivery is experienced by participants could inform any adaptations needed for online delivery of MBPs and help support wider access.

**Methods:**

Data from 159 MBP participants were collected via a 46-question online survey. Responses were analysed using SPSS. Content Analysis was undertaken on the qualitative responses.

**Results:**

Participants were generally satisfied with MBPs delivered online, and their MBP teacher, and identified both drawbacks and benefits to learning online. Although online offerings seem to have broadened access, MBP participants were not diverse across all demographic measures. Participants welcomed the opportunity to learn MBPs through online methods, appreciating practical advantages and the internationalism of their groups. Nevertheless, many reported a felt loss of connection with others in the group, and high levels of distractibility. Many participants (28%) said they did not have any type of pre-course orientation.

**Conclusions:**

It may now be timely for the field to review experiences of online MBP teaching and learning to identify areas where online implementation may have been constrained by in-person theory and practice, and what helpful adaptations could be made to both curricula and practice. This could include developing specific guidance around managing online distractibility, and how to best facilitate the group process online.

## Introduction

In the almost half century since Jon Kabat-Zinn introduced Mindfulness-Based Stress Reduction (MBSR) to a clinical setting in North America, people around the world have experienced significant mental, physical and psychological health benefits from participating in Mindfulness-Based Programs (MBPs) [1]. MBPs have become more extensively available, particularly in the West, and there are many adapted MBPs to support those facing specific psychological and physical challenges, such as Mindfulness-Based Relapse Prevention [2, 3] and Mindfulness-Based Eating Awareness Training [4, 5]. MBPs are defined by the British Association of Mindfulness-Based Approaches (BAMBA) as having a set curriculum informed by a clear rationale with a commitment to being evidence-based; led by a teacher, they involve incremental development and experiential learning, usually over at least eight sessions, and include a daily meditation home practice commitment [6]. A review of MBPs [1] identified that they were efficacious in improving many biopsychosocial conditions (such as depression, anxiety, insomnia, addiction, psychosis, pain, hypertension, weight control, and cancer-related symptoms). MBPs were found to be relatively safe, and preliminary evidence suggests cost-effectiveness in some contexts (for example breast cancer, fibromyalgia, low back pain, and caregiver training). Evidence was inconclusive or only preliminary for the effects on some other conditions (e.g. eating disorders, loneliness, and diabetes) and the quality of many included studies was questionable, particularly with regards to small sample sizes and short follow-up periods.

During the pandemic there was a rapid switch to online MBP teaching; online MBPs are now routinely offered and highly accessible [7]. In the several years since the global pandemic, research into efficacy of online MBPs has substantially increased; a systematic review [8] showed that online MBPs had a significant low-to-moderate impact on mental health, and that these effects were maintained at short-term follow-up. When examining participant outcomes for those experiencing symptoms such as depression and anxiety, the impact of online MBPs appeared similar to MBPs delivered by traditional face-to-face format, irrespective of the type of online MBP offered.

However, prior to the global pandemic and beginning of international lockdowns or restrictions early in 2020, live, synchronous online delivery of MBPs was the exception rather than the norm. The reasons for this are likely multifactorial and perhaps a consequence of the complexity of mindfulness teaching and learning. In a review of essential resources for mindfulness teachers [9] an important distinction is made between the explicit MBP curriculum, and the implicit. It seems that mindfulness teachers in training appear to first master the explicit aspects of delivering the MBP curriculum, such as guiding practices and managing the logistics of teaching. Only once competence has been acquired in these realms, are teachers then able to cultivate the more intuitive, embodied aspects of the teaching process—the implicit curriculum—which includes embodiment (being aware of and present with current physical and mental states), relational skills, and group process. It is further suggested that teaching capacity in the implicit curriculum is what enables the teacher to catalyse participants’ growth and transformation. Given the subtleties described, which are inherent in mindfulness teaching and learning, it seems possible that both conveying and receiving the potentially transformational curriculum, particularly the implicit components, may be significantly affected when the delivery medium moves from in-person to online. As online delivery now appears to be mainstream [10], it seems important to understand participants’ experiences, to identify any gaps in the curriculum that are conveyed online and to consider scope for improving online delivery of MBPs. One study identified that military veterans developed greater trust during an in-person MBP when compared with online [11], findings that endorse our interest in participant experiences more generally. A study about Zoom interactions versus in-person showed a disparity in neural activity, with less neural activity linked with gaze time, face processing, and pupil dilation during zoom interactions [12].

### Telehealth experiences

We were unable to identify any literature which specifically explored participants’ general experiences of attending an MBP online. The papers generated by our searches were highly nuanced and typically related to specific populations and were not directly comparable with our broader objectives; therefore, we considered research into experiences of telehealth – a means of experiencing healthcare services via electronic devices (live audio and video appointments as well as secure text messaging from healthcare providers) [13]. This exploration of the wider literature seems helpful because telehealth shares much in common with online MBPs, specifically regarding mode of delivery, its rapid upscaling during the pandemic [14] and the common overarching intention to alleviate suffering. Like telehealth, some MBPs are therapeutic, (e.g. MBCT for recurrent depression [15, 16]) and others psycho-educational, (e.g. MBSR for general populations [17, 18]) and so the findings of studies into experiences of telehealth seem likely to offer insight as to what might be experienced when MBPs are delivered online. We therefore considered studies looking at clinicians’ experiences of telehealth, followed by those of patients who have accessed treatment remotely.

Telehealth efficacy was considered by many clinicians to be equivalent to face-to-face care [19]. Clinicians’ perceptions of the rapid scale-up of telehealth services led researchers to conclude that telehealth was feasible and effective for treating depressive and anxiety disorders [20], whilst clinicians also considered telemental heath a valuable tool for treating mental health and substance use disorders [21]. These early studies into telehealth efficacy suggest that a shift to online delivery has enabled an acceptable level of program efficacy to be achieved in clinical services.

Clinicians felt they could establish rapport and meet clients' needs through telehealth, though when asked to compare telehealth with in-person treatment, only a minority felt that telehealth met their clients’ needs equally well or better. Other research describes clinicians’ resistance to perceived challenges of telemental health, specifically fears around difficulties in building therapeutic alliance in telehealth settings, when compared with in-person settings [20]. Challenges in establishing rapport and engaging clients in a telehealth group setting were also noted [20].

Despite initial reservations, telehealth clinicians adapted to the online modality, learning different ways to build rapport with clients [21]. The digital environment brought specific challenges as well as strengths, and it seemed that specific telemental health training, particularly relating to cultural humility and alliance building, is important, particularly because broadened accessibility may attract clientele who were not coming to therapy until telehealth was available. The findings in these studies offer consensus that telehealth therapies impede the building of important relationships between clinician and service users, compared with in-person delivery, and it seems likely that these considerations may be relevant to online MBPs, where rapport between teacher and participants is also particularly important [22] and cultivation of relationships both with the teacher and within the group, key elements of the implicit curriculum, could be impacted by a change in program modality.

A study into provision of cognitive behavioural group therapy via videoconferencing [23] reported that although telehealth services removed transportation barriers, digital delivery presented new challenges, such as the need for a safe and private location for clients to engage with the program. Technical and clinical challenges were also encountered, including difficulties in seeing and hearing participants effectively. Again, these observations seem transferable to the online MBP setting.

As outlined above, the existing literature identifies some consistent themes concerning telehealth and MBP professionals’ experiences of supporting others online, though how participants experience the learning online is less clear. Although there is a plethora of acceptability studies of online MBPs [24] and telehealth [25, 26], these report little beyond data on participants' general satisfaction with the experience. There is a dearth of literature exploring participants’ experiences of online MBPs and relatively little in the wider telehealth literature about how participants directly experience telehealth. One of the few studies that explores participants’ felt experience of telehealth [14] reports that clients felt a loss of the sense of "groupness" when not physically present in the same room for the group components of Dialectical Behavioural Therapy. This is a relevant consideration for participants engaging in online MBPs, which are also typically delivered to groups. Furthermore, group process has been identified as an important ingredient in MBP participants’ learning, a component of the implicit curriculum, where the sharing of communal experiences and the felt connectivity with other group members enhances learning for all [27–29]. It would therefore be helpful to understand how MBP participants perceive their experience of the group when engaging online, and to consider whether online-specific adaptations are required to optimise felt group cohesiveness.

It has been suggested that more sensitive topics are more often raised in the telemental health setting, perhaps because the physical distance creates a degree of comfort and being behind a screen creates a deeper sense of safety [21]. Challenges relating to the group, privacy, technology, and managing sensitive subjects seem very likely to also be encountered in the online MBP group setting, and we would therefore like to understand online participants’ experiences of these potential difficulties when learning online.

MBPs are complex interventions, and an online context may present challenges that reduce their effectiveness. For example, it has been noted [14] that in the telehealth setting, non-verbal communication may not translate well over technology, leading to increased anxiety for telehealth clients. Again, this possible online impediment to communication is highly relevant to mindfulness trainings where the intention is for participants to ‘catch’ the mindfulness that is modelled by the facilitator via implicit learning [30]. If non-verbal cues are clouded by the delivery medium, this could also impact sharing of the implicit curriculum.

A systematic review into the enablers and barriers to participation in online MBPs amongst informal carers [31] found that online delivery of MBPs could be suitable for supporting carers who could not attend face-to-face programs. Self-motivation, previous positive experiences of meditation, program structure and delivery were identified as key enablers and barriers to engaging with online MBPs, and it seems likely that these findings may be generalisable to other populations accessing MBPs. Given the diversity of physical and mental health conditions that may be alleviated by participation in an MBP and the potential for their scalability due to greater accessibility via online delivery, it seems important to gain greater understanding about what it is like for participants to attend an online MBP. It would be particularly helpful to know whether the experience of participating in an MBP online is generally satisfactory, how the online group or teacher is experienced, and to identify any aspects of the program that could be further enhanced to suit the online modality.

### MBP participant demographics

Since online delivery of mindfulness has made MBPs much more broadly available globally it seems pertinent to also consider who attends online MBPs. When the systemic barriers that impede access to MBPs are considered it seems that there is a need to make mindfulness trainings and programs more equitable and accessible to all, regardless of socioeconomic status, ethnicity, or ability [32]. Demographic findings obtained in an efficacy study of in-person mindfulness suggest that many marginalised groups are under-represented [33]. Of their sample of 127 participants, 84% were Caucasian, 74% female with a median annual income of $150,000. In our experience, this is typical of populations accessing mindfulness training and so, as some significant barriers to access (e.g. transport, costs) will have been mitigated by the increase in online MBP delivery, there is a need to know who is accessing online mindfulness.

This study is exploratory, and its aims include: 1) to gain understanding of participants’ experiences of learning MBPs online, particularly with respect to pedagogical aspects, 2) to explore the demographics of MBP participants to understand who is accessing MBPs online.

## Methods

### Participants

A total of 161 participants were recruited. Inclusion criteria were: 1) over the age of 18, and of any gender, or geographic location, and 2) to have attended at least one session of an online eight-week MBP which was taught synchronically by a teacher in a group setting. We utilised the Crane et al. definition of an MBP [30] to determine inclusion/exclusion of a particular MBP curriculum, including only responses from participants who had joined courses that maintained fidelity to what Crane et al. defined as essential MBP components [30]. Recruits were eligible to participate even if they had not completed the MBP as we wished to capture reasons for non-completion of the program, and to not exclude those who may have been dissatisfied with their experience.

Participants were recruited via one of two routes; the first was a social media campaign and the second was direct contact with international mindfulness training centres asking them to share information about the study. The social media recruitment campaign was launched on Facebook and X (formerly Twitter) and via ‘snowballing’ where we invited account followers to share survey information and links with their own mindfulness contacts. We directly emailed MBP teacher training organisations and associations of MBP teachers in the United Kingdom (UK) and internationally, requesting that they share study details and the link to the survey with their mailing lists or newsletters and social media. We provided advertising imagery and narrative. Because the survey was shared and re-shared by multiple contacts, it is not possible to determine the response rate. As there is no data on how many people have taken an MBP course online, it is unknown what percentage of MBP participants engaged with the recruitment campaigns.

### Procedure

Ethical approval for this project was granted by Bangor University School of Psychology Ethics and Research Committee in August 2021. To recruit participants, a social media outreach campaign was launched**,** which provided the survey link. Participants were asked to read the information sheet and could not access the online survey without first consenting on its opening page. No financial incentive was offered for participating in the survey. All survey questions were optional so that they could be omitted if the participant preferred not to answer. The survey opened on 8 February 2021 and closed on 30 April 2022.

The survey was designed by the research team who are also MBP trainers and teachers with experience of delivering teaching and training online. A focus group for usability testing was set up. The draft survey was shared with 18 experienced MBP teachers who have taught online, and they were invited to feedback on the usability and content of the survey. Eight of these teachers subsequently met with the authors to evaluate the survey line-by-line to check for clarity and relevance of the questions and no substantive changes were proposed. The group suggested some minor adjustments to enhance clarity and to ensure that the survey was appropriate for its intended audience. The types of changes made were adjusting open-ended questions to a multiple-choice format or adding space for clarifying comments. This survey test ensured that the questions addressed the aims of the study and could also be completed within a short timeframe. All proposed amendments were adopted. There was a total of 21 qualitative questions included in the survey. Of these, 11 were standalone questions, whilst the remainder offered the participant space to comment further on their response to the quantitative component of the question. The survey was designed to take around 20 min to complete and opened with 12 multiple-choice questions about the participants’ demographics (e.g. gender, age, ethnicity, health, residence, household income, highest level of education, religious beliefs). These were followed by 11 questions about their practical experiences of participating in an online MBP, for example which type of MBP they did, the number of sessions joined, and any technological challenges experienced. The next four questions considered other aspects of learning mindfulness online, such as the home environment. These and subsequent questions were a combination of quantitative multiple-choice and related qualitative open-ended questions which enabled participants to supplement an answer with a comment if they chose to do so. Participants were then asked to consider seven further questions relating to their interaction with course teachers and then eight questions about experience of the group. Three further questions explored participants’ views of advantages and disadvantages regarding the MBP online format, and any other observations.

The risk of distress to survey participants was considered minimal as the questions focused on their practical and felt experiences of the online MBPs. To safeguard respondents, survey participants were given the researcher’s contact details and invited to contact them if they experienced distress during or having undertaken the survey. No participants contacted the researcher for this (or any other) reason.

### Data analysis

IBM SPSS (Version 27, being current at the time) was used to analyse the quantitative data, and the data set was cleaned and adjusted in the following ways: (i) two participants wrote that they paid £4,000 for their MBP and a third said that it cost £3,500. These figures were excluded from the data due to the strong likelihood of them being typing errors; (ii) two participants reported that their MBP was delivered via pre-recorded sessions and so we excluded their data. Where financial information was provided in other currencies, fees were converted to British Pounds (GBP) using an average of the rates in effect during the survey period.

Every comment shared in response to the open-ended questions was coded using content analysis to identify patterns in the data. Qualitative content analysis aims to identify specific themes or patterns in the dataset by identifying codes and refining the emerging system to enhance relevance and credibility [35]. The researchers considered their own biases regarding these responses (for example, ensuring that critical comments were treated in the same way as positive comments) and took care to treat all data equally. All comments were saved into separate master spreadsheets. The content was reviewed to identify the categories that would be used for coding that specific question, so that an inductive coding scheme could be established based on survey contents rather than researcher expectations. From here, comments were copied line-by-line into a separate Word document for each question and coded according to the primary theme of the sentence. In some instances, it became apparent that additional and sub-themes could be identified, necessitating development and refinement of the coding scheme. (For example, an overarching reason for participants having missed a session might have been a change of circumstances and within that, several participants may have indicated that they experienced bereavement during the course, whilst others may have referenced work commitments, and so these sub-themes were identified to further refine the coding process). Comments were typically a sentence in length, which meant that identifying the key theme was usually straightforward. Where a respondent made several remarks in the qualitative component of a question, the content was placed into all applicable content categories. For example, within the open-ended question about perception of the diversity of other people in their group, a participant may have made remarks about ethnicity, age, and gender and so their response would have been allocated to each of those subcategories. This means that where a content analysis table shows *N* total responses to a question, it is probable that fewer participants answered the question, and that some offered observations relating to multiple themes within each answer. The third author reviewed and triangulated the data on a sample basis and was satisfied that their inter-coder reliability tests indicated a consistent approach to data analysis. There were no disagreements concerning categorisation of themes.

## Results

### Demographics of survey participants

Of the 159 survey participants included in the study, 83.1% identified as female, 16.2% as male and 0.5% as non-binary. The mean age was 48.16 years (*SD* = 12.39). Of the participants who disclosed their race (87%), seven ethnic categories were identified, with 71.9% of participants describing themselves as White or Caucasian. The next largest group was Asian (10.5%), with much smaller groups of two to four people identifying as each of Arab, Black, Hispanic, Latin and Mixed Race. Course participants were located across 26 countries. Of these, the majority (81.2%) were based in Europe, of which 61% were resident in the UK though responses were also received from participants living in China, Hong Kong, Indonesia, Japan, Lebanon and USA.

The survey participants reported a high level of education: 90.1% of those who answered the question had graduated from university, whilst 51.8% of these had a postgraduate qualification. A number of mindfulness course attendees (40.7%) were in full-time employment. As outlined in Table 1, household income data showed a range of income levels, 69% of participants stated that their total household income was more than £30,000 per annum whilst ONS reports that median household income after direct taxes have been deducted in the UK was £31,400 in financial year ending 2021 [34] suggesting that this population is more affluent than the average British household.

**Table 1:** Participant background

	*n*	%
**Highest Academic Qualification**	*N:*	156
Non-graduate	15	9.6
First degree^a^	60	38.5
Postgraduate degree^a^	81	51.8
**Employment Status**	*N:*	150
Employed full-time	61	40.7
Employed part-time	40	26.7
Retired	20	13.3
Student	12	8.0
Unemployed	17	11.3
**Household Income (GBP)**	*N:*	132
Under £20,000	25	18.9
£20,000-£29,999	16	12.1
£30,000-£49,999	31	23.5
£50,000-£69,999	21	15.9
> £70,000	39	29.6
**Ethnicity**	*N:*	153
Arab	*3*	2.0
Asian	16	10.5
Black	2	1.3
Caucasian	110	71.9
Hispanic	1	0.7
Latino	2	1.3
Mixed race	4	2.6
Unspecified	15	9.8
**Residence**	*N:*	150
Australasia	1	0.7
Asia	12	8.0
Europe^b^	129	86.0
N America	7	4.7
S America	1	0.7
**Religion**	*N:*	124
Atheist/Agnostic	47	37.9
Buddhist/secular	11	8.9
Christian	40	32.3
Humanist	4	3.2
Muslim	2	1.6
Spiritual	9	7.3
Unsure/Other	11	8.9

^a^or equivalent qualification

^b^Of which UK: *n* = 97 (61.0%)

Of the 159 survey participants, 35 (22%) did not answer the question about religion and a further 58 (36.4%) reported they were either unsure about religion or affirmed non-believers. Of the total number surveyed, 25.2% reported they were Christian and 6.9% described themselves as Buddhist or having Buddhist leanings.

### Mindfulness course experience

Participants’ online courses had typically been delivered within the seven to twelve months prior to the survey (40.5%) with 36.8% having been taught online within the last six months. The mean fee paid (excluding three outliers) was £245.04. (SD = £151.59, Range = £0-£1000), with 6.9% of participants having offered their course free of charge (Table 2).

**Table 2:** Online MBP attended

**When Online MBP Completed**	*n*	%
Within last month	12	7.6
Within last 2–3 months	32	20.3
Within last 4–6 months	26	16.5
Within last 7–12 months	64	40.5
Other	24	15.2
**Most Recent Online MBP**^a^	*N:*	157
MBCT-L	83	52.9
MBSR	37	23.6
MBCT (D)	19	12.1
Advanced	5	3.2
MBChP	5	3.2
Other	3	1.9
Breathworks	2	1.3
Compassion	2	1.3
FPFW	1	0.6
**Course Cost (GBP)**	*N:*	115
Free of Charge	11	9.6
£1-£49	2	1.7
£50-£99	2	1.7
£100-£149	5	4.3
£150-£199	10	8.7
£200-£249	16	13.9
£250-£299	39	33.9
£300-£399	22	19.1
£400-£499	1	0.9
£500 +	7	6.1

^a^*Abbreviations* as follows: *MBSR* Mindfulness-Based Stress Reduction, *MBCT (D)* Mindfulness-Based Cognitive Therapy (Depression), *MBCT-L* (Mindfulness for Life) Compassion – Mindfulness-Based Self-Compassion and Mindfulness-Based Compassionate Living, Adaptation—teachers’ own adaptations, Mindfulness-Based Childcare and Parenting, Health – includes Mindfulness programmes for addictions, eating disorders, COVID19, Breathworks, general health conditions; *FPFW* Finding Peace in a Frantic World, Advanced – Deeper Mindfulness and Taking it Further

Most of those who engaged in the survey said that they were extremely (67.3%) or moderately (28.9%) technologically competent. The majority (61.0%) reported no technological issues but of those who experienced problems, the most frequently occurring of these was internet disruption – 10.7% had encountered this difficulty during their online course. The majority of participants (148) accessed their MBP via the Zoom platform.

Attendance is outlined in Table 3: only 2% of participants said they missed more than three sessions, whilst the majority (87.2%) attended all sessions of the MBP. 61.3% of participants thought their group was somewhat diverse and 34.2% reported that their group was extremely diverse, when considering factors such as ethnicity, occupation, gender, and age. Pre-course group meetings had been attended by 13.2% of participants whilst 13.9% had spoken to teachers on a one-to-one basis in advance of the course starting. A substantial 22.6% had not been asked to provide any personal information prior to attending the online MBP.

**Table 3:** Experiences of Online MBPs

	*n*	%
**Sessions Attended**	*N:*	153
All	128	83.7
Missed one	17	11.1
Missed two	3	2.0
Missed three or more	3	2.0
All, so far	2	1.3
**Number of Course Participants**	*N:*	101
1	4	4.0
2–4	3	3.0
5–9	23	22.8
10–14	41	40.6
15–19	22	21.8
20–24	5	5.0
> 25	3	3.0
**Diversity of Online Group**	*N:*	155
Extremely Diverse	53	34.2
Somewhat Diverse	95	61.3
Not at all Diverse	7	4.5
**Pre-course Orientation**	*N:*	159
Group Online Meeting	21	13.2
One-to-One Meeting	13	8.2
Telephone Call	9	5.7
Information Gathered	36	22.6
None	45	28.3
Other	5	3.1
More than one of these	30	18.9

Participants were provided with opportunities to expand on their experiences of learning mindfulness online in a series of open-ended questions. Table 4 summarises the themes and subthemes identified.

**Table 4:** Content analysis—experiences online

**Perceptions of Diversity**			
*N*: 145	*n*	*n*	
Diverse:	Diverse	Non-Diverse	
Age	15	7	*'Ages ranged between around 30 and 70'*
Culture	3	1	*'many diverse cultural backgrounds'*
Ethnicity	9	13	*'I think everyone was white bar one person'*
Gender	15	10	*'gender was 50% male 50% female'*
Health/Ability	4	4	*'It was a group of individuals with a specific diagnosis'*
Marital Status	2	0	*'different marital status'*
Nationality	36	2	*'It was lovely to learn with people from all over the world as they brought different perspectives and experiences'*
Occupation	11	5	*'Occupation: great variety, including retired'*
Religion/Beliefs	2	0	*'1 muslim lady'*
Sexuality	0	2	*'Mostly hetero couples'*
Social Class	0	2	*'Mainly what looked like middle class'*
Other Comments		*2*	*'It was interesting to see how we all had different points of views when we were given same scenario'*
**Reasons for Non-attendance**			
*N*: 23	*n*		
Time Constraints			
*- Work*	4		*'Night shift work and sleeping the next Day. On course day-time.'*
*- Family*	3		*'I had a family event which ran over and I didn’t want to leave'*
*- Travel*	7		*'Trip away from internet access'*
*- Other*	1		*'Appointment'*
**Change in Circumstance**			
*- Family*	1		*'Family emergency'*
*- Bereavement*	1		*'family bereavement'*
*- Illness*	4		*'Got covid!'*
*Not motivated*	2		*'the last thing I wanted to do was to spend more time on zoom but I also felt the energy of the sessions was really lethargic and clinical'*
Home Set-up Guidance			
*N*: 88	*n*		
***Guidance*** **concerning:**			
- *Environment*	31		*'Be in a comfortable, private setting without distractions'*
*- Technology*	11		*'Lighting and house keeping, staying in the frame, etiquette'*
*- Equipment*	2		*'Guidance on bringing a mat, bringing a beverage, and having access to layers of clothing'*
No Guidance given	18		*'I don't remember there being specific instruction'*
Delivery Format	18		*'The course joining instructions provided some helpful guidance'*
Miscellaneous	8		
**Camera Use Guidance**			
*N*: 113	**n**		
Cameras always on	*62*		*'Keep cameras on all the time, at first I found this confronting, but it made sense the more I continued in the program'*
On, except during practice	9		*'We were encouraged to leave our camera on as much as possible except during the meditation exercises'*
Optional	23		*'Use of camera was optional according to individual needs. Nice to have the flexibility'*
No guidance given	5		*'No specific instructions relating to use of cameras'*
Forgotten	5		*'Can’t remember..we all had our cameras on. Nobody ever switched them off'*
Miscellaneous	9		

Few participants expanded on the question about their own technological difficulties (*N* = 9). The themes identified were use of the ‘chat’ function (*n* = 2), sound (*n* = 2), with miscellaneous responses making up the remainder.

When asked about perception of participant diversity online, 11 sub-themes were identified. These were: age, culture, ethnicity, gender, health/ableness, marital status, nationality, occupation, religion, sexuality, and social class. Of 145 responses, 97 commented that their group had consisted of people displaying diverse traits whilst 48 observed a lack of diversity. The most common response (*n* = 36) was that they felt there was a wide geographical diversity, as well as age (*n* = 15), gender (*n* = 15), and occupation (*n* = 11). Some participants considered that there was a lack of diversity in terms of age (*n* = 7), gender (*n* = 10), and ethnicity (*n* = 13).

Participants’ attendance was generally good and so only 23 people commented further on reasons for lack of attendance. These were split into the broad categories of time constraints (*n* = 15), change in circumstances (*n* = 6) and having felt somewhat unmotivated (*n* = 2).

Many participants (*N* = 88) reported having received guidance from the MBP teacher about how to set up their home environment. Of these, 31 mentioned the space itself, with descriptions such as ‘quiet’, ‘comfortable’ and ‘undisturbed’ occurring frequently. A further 11 reported receiving guidance concerning technology and two mentioned the equipment they had been advised to access. Another 18 said they could not recall having received any guidance.

When asked to describe the guidance they received from their teacher regarding having their cameras on or off during online teaching and if/how this impacted on teaching and/or sense of the group, 113 participants responded. Most (*n* = 62) reported that they were asked to always keep their cameras on, with a further nine participants saying they were permitted to turn them off during practices. Another 23 participants interpreted the guidance offered as camera use being optional. Additionally, six people stated that no guidance had been given, five more had forgotten and a further nine made miscellaneous comments.

### Participants’ experiences of attending an online mindfulness course

The survey asked course attendees to consider pedagogical aspects of their online learning experiences such as their commitment to the course, experiences of distractions from the home environment and overall experience of being taught via an online medium. In general, most felt their online learning experiences were successful: 42.8% said that the course had met their expectations whilst 55.3% found that the course exceeded their expectations. Participants also reported strong levels of commitment to the program: 79.2% of participants described themselves as extremely committed. Over half (54.8%) felt a strong connection with their instructor and of the remainder, almost all (a further 41.3%) said they had felt some connection with the teacher.

The picture is similar regarding feelings of being supported and safe in the online learning environment (Table 5), which shows that the overwhelming majority found the experience to be supportive and safe enough to disclose personal information. More than half of participants (56.1%) found their groups to be somewhat open regarding disclosure of personal experience (they perceived that others felt willing to share their experiences), whilst a further 42.6% found their groups to be extremely open.

**Table 5:** Commitment, Connection and safety

	*n*	%
**Overall Experience of Online MBP**	*N:*	159
Exceeded Expectations	88	55.3
Met Expectations	68	42.8
Did Not Meet Expectations	3	1.9
**Commitment to Online MBP**	*N:*	159
Extremely Committed	126	79.3
Somewhat Committed	32	20.1
Not at all Committed	1	0.6
**Feelings of Connection with Instructor**	*N:*	155
Extremely Connected	85	54.8
Somewhat Connected	64	41.3
Not at all Connected	6	3.9
**Feelings of Connection with Group**	*N:*	155
Extremely Connected	37	23.8
Somewhat Connected	103	66.5
Not at all Connected	15	9.7
**Feeling Supported by Teacher**	*N:*	157
Extremely Supported	114	72.6
Somewhat Supported	42	26.8
Not at all Supported	1	0.6
**Level of Disclosure in Group**	*N:*	155
Extremely Open	*66*	42.6
Somewhat Open	87	56.1
Not at all Open	2	1.3
**Participants' Feelings of Safety in Group**	*N:*	159
Extremely Safe	*106*	66.6
Somewhat Safe	51	32.1
Not at all Safe	2	1.3

Having responded to the initial quantitative questions, participants were invited to comment regarding aspects of their experience such as feelings of commitment to the program, descriptions of the MBP teacher and how the teacher had made them feel, connection, support and inclusion. See Table 6 for themes and sub-themes.

**Table 6:** Content analysis – commitment, teacher connection and support

**Experience of Online**		
*N*: 53	*n*	
Online MBP was Positive	27	*'Was really impressed with how well the course could be delivered online. The mixture of talk & time to practice plus the collaboration exercises were important I think.'*
MBP (in general) was Positive	12	*'Course is of exceptionally high standard. It really helped me re engage with mindfulness (a primary goal) and helped me re centre my thinking.'*
Prefer In-person, Online Adequate	5	*'I would still prefer in person, if possible, but I got much more out of doing the course online than anticipated.'*
Online MBP was Negative	4	*'extremely difficult to engage with because it seemed to be an attempt to literally translate of what would otherwise happen f-2-f, without controlling for online environment.'*
MBP was Negative	2	*'Did not enjoy'*
Miscellaneous	3	*'Competence and personality of the teacher are important for me.'*
**Commitment**		
*N*: 53	*n*	
Very Committed	21	*'I did all the practices including the home practices on a regular basis (every day of the week until the next session)'*
Positive Experiences	11	*'It changed my life'*
Moderately Committed	9	*'Time needed to practice was huge, I did my best'*
Not Committed	3	*'I had too much on my plate so lacked the time and focus'*
Negative Experiences	2	*'The online sessions lacked the sense of connection with community.'*
Miscellaneous	7	
**Connection with Main teacher**		
*N*: 34	*n*	
In-Person/More 1–1 Would Help	12	*'We never had a personal conversation and I think this would have made me feel more connected'*
Teacher Facilitated Connection	7	*'She really put a lot of energy and effort into the sessions, she explained her own process using her own experience'*
Other Positive Remarks	5	*'Both teachers where very engaging but I actually formed more of a bond with the second facilitator as we did more small group work together'*
Responsive to Contact	3	*'I asked questions by email and he responded helpfully and in detail'*
Very Connected	3	*'I am not putting "extremely", because to me it borders dependency:) But I felt very much at ease and in tune with the teacher.'*
Miscellaneous	4	*'I don't know the guy, pure and simple'*
**Support**		
*N*: 32	*n*	
Good Support from Teacher	13	*'Our teacher was very open, responsive and supportive. He always made me feel as though he wanted to help if he could'*
Opportunities for Support	10	*'Any questions I had were answered promptly online or email'*
Support from Teacher Lacking	3	*'There wasn’t much individual connection'*
Miscellaneous	6	*'I didn't ask for an extra mile so far, so I don't know if he would run it for me'*

When asked to comment further about their experience of online MBPs, 52 comments were received. The majority commented that learning an MBP online had been positive (*n* = 27) with a further 12 commenting favourably about MBPs more broadly, without reference to the delivery medium. A further five participants commented that online delivery was adequate, but that in-person would have been preferable. Of those who enjoyed the experience less or not at all, four referenced online MBPs and two talked about the program more generally. There were three miscellaneous comments.

Additional comments about felt commitment drew 53 responses. Of these the majority (*n* = 21) were very committed, whilst 11 talked about their positive experiences. A further nine considered themselves moderately committed to the program whilst three had struggled to commit and two comments were about negative experiences of the course.

**Fig. 1:**
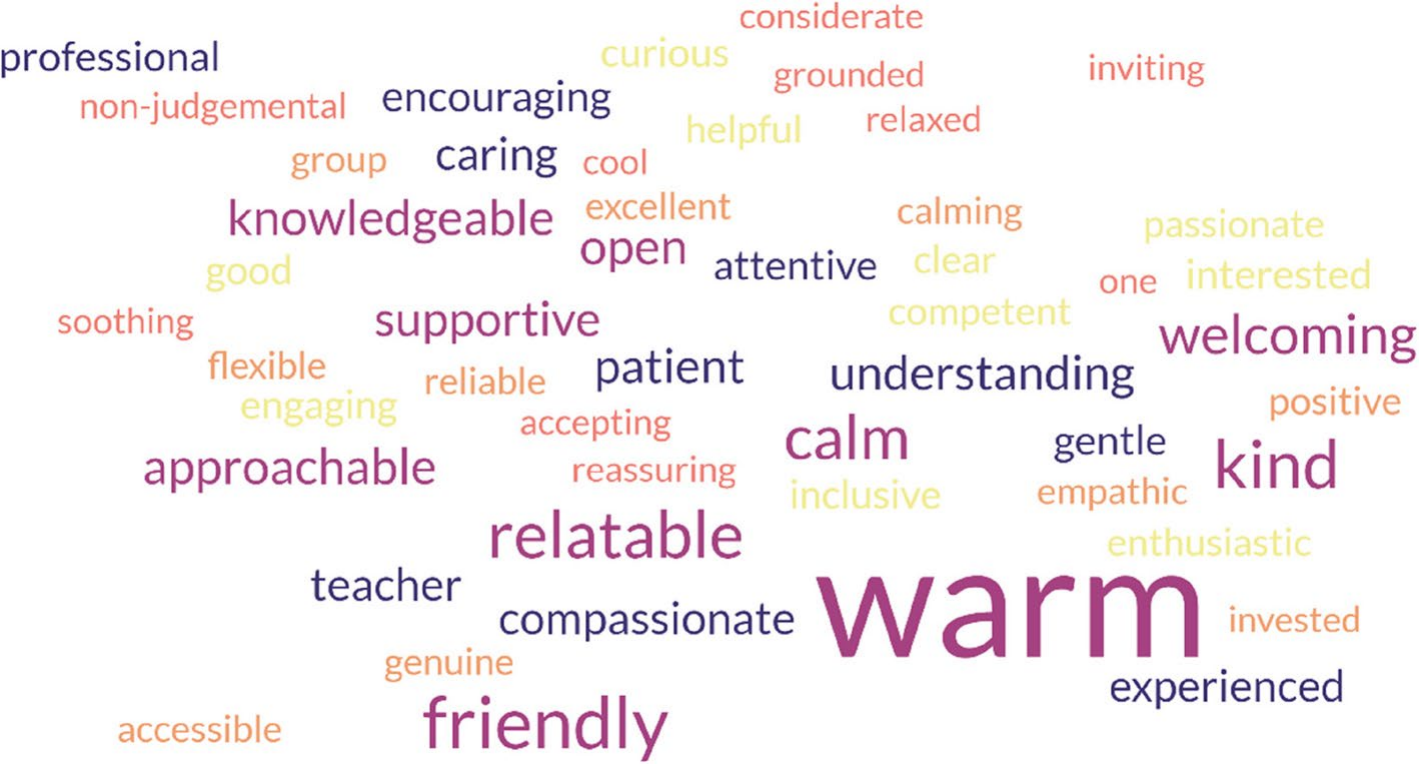
How participants described their teacher

There were many responses (*N* = 137) to the question which asked participants to write up to five words describing the attitudes of the MBP teacher. The most effective visual way of reflecting the frequency of the adjectives used was to generate a Wordcloud [36]. As shown in Fig. 1 below, the most commonly recurring word, identified 96 times (by 60.3% of participants), was ‘warm’. Then, ‘approachable’, ‘calm’, ‘friendly’, ‘knowledgeable’, ‘open’, ‘relatable’ and ‘supportive’ were the next most frequently used descriptions. All the words chosen had positive connotations.

**Fig. 2:**
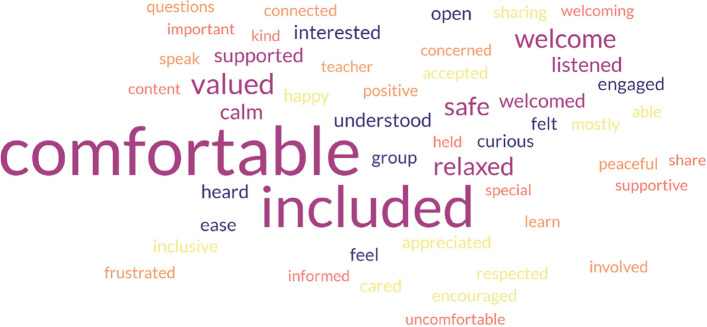
How participants said their teacher made them feel

Similarly, when asked how the teacher made participants feel, the responses (*N* = 134) were largely positive (Fig. 2), with ‘comfortable’ the most frequently occurring (*n* = 124) description used by 78.0% of participants, followed by ‘included’ ‘valued’, ‘safe’, ‘welcome’ and ‘relaxed’. Again, the words chosen were diverse and almost invariably positive, though ‘uncomfortable’(*n* = 2) ‘frustrated’(*n* = 1), ‘occasionally not listened to’ (*n* = 1) and ‘hurt when muted’ (*n* = 1) were also written.

Of the 34 participants who commented about their feelings of connection with the main teacher, most (*n* = 12) felt that being in-person or having more one-to-one time would have been beneficial. A number (*n* = 7) noted that the teacher had facilitated connection and five others made general positive comments. Several (*n* = 3) participants commented that the teacher had been responsive to contact and three said they felt very connected to their MBP teacher.

Responses (*n* = 32) regarding the extent to which participants were supported by their teacher were similarly positive to those about feelings of connection. Most of these (*n* = 13) felt that they had received good support from their teacher; a further ten commented that opportunities for support had been made available. A minority (*n* = 3) had not felt well-supported, whilst six others made miscellaneous comments, such as not having needed support so finding this difficult to assess.

There were 25 responses to the question about feelings of inclusion within the group. It was not possible to identify recurring themes here as the comments were disparate and only a handful relevant to the theme of inclusion; being fully accepted in the group and treated equally. Most strayed into commenting on the quality of connection with the group online, which was specifically covered elsewhere within the survey, though several did comment on not having felt included, or having felt included but that this had not been the same as experiencing a sense of belonging.

### Participants’ distractibility and experiences of group connection

When considering the extent to which participants experienced distraction during their online MBP, most participants (59.4%) reported having been somewhat distracted. A further 35.6% claimed to not have experienced distraction whilst 4.4% found themselves significantly distracted. The types of distractions mentioned included children and other family members being at home, the doorbell ringing, and activity from pets.

The survey asked participants to share the opportunities that were made available to connect with other group members. Of these, the most commonly occurring was chat function enablement (10.1%), WhatsApp group (10.1%) and opening the session early for informal chats (9.4%) whilst 38.4% of participants experienced multiple different opportunities to connect. A significant number of participants (*n* = 29, 17.6%) did not experience any opportunities outside of the MBP sessions to get to know fellow group members.

The use of breakout rooms (small ‘rooms’ within a meeting enabling a specific group of participants to meet) was explored, with 137 participants reporting that they were used regularly during the MBP. The majority (53.3%) had usually worked in pairs and 85.4% reported that the members of their breakout groups changed. The experience of breakout rooms was considered enjoyable by 78.8% and 82.5% felt comfortable. Staying on task was not always easy with only 52.6% of participants saying they managed this, whilst teacher visits into breakout rooms were rare—19 participants (13.9%) reported that their teacher had done this.

Participants were invited to comment further on the nature of their informal interactions, group connection, safety, and openness—the themes identified are summarised in Table 7.

**Table 7:** Content analysis: experiences of the group

**Informal interaction**		
*N*: 56	*n*	
Available During Course	23	*'Every session started with about 5–10 min of socializing and what’s app was used as needed by participants between sessions'*
Set up After Course	13	*'We came to know one another quite well and supported each other, We set up a WhatsApp group towards the end of the course'*
Nothing Offered	7	*'None of the above and I really think that some more opportunity to connect with everyone would have been very welcomed'*
Didn't Engage	3	*'During breaks people could socialise- but to be honest I needed the screen break so I didn't take this up.'*
Miscellaneous	13	*I didn't think the lack of 'social' stuff was a bad thing. We got plenty of interaction in the breakout rooms anyway*
**Group connection**		
*N*: 40	*n*	
Lacking Connection	14	*'Normally, in a group like that, I would have felt like I wanted to be in touch with at least someone, but I didn’t feel that connected to anyone'*
Adequate Connection	11	*'I think that we connected very well considering that it was online, but stronger connections would have been made if we had been in the same room '*
Positive Connection	11	*'Was surprised how much I grew to be like them and quickly formed a group'*
Miscellaneous	4	*'We mostly knew each other before'*
**Group Safety**		
*N*: 26	*n*	
Safe to Share	13	*'We all were prepared to share difficulties in our life and there was a lot of comfort and affirmation from others'*
Not Safe to Share	5	*'Found it difficult to talk having never met any of the participants'*
Neutral Remarks	3	*'none seem interested in me as a person, which is good in these cases'*
Miscellaneous	5	*'Expressing myself is always a challenge for me, its not that I wouldn't have felt safe'*
**Group Openness**		
*N*: 29	*n*	
Some Openness in Group	18	*'We shared safely about our difficulties but not to an extend where we would feel emotionally overwhelmed or exposed. it was very balanced.'*
Group Didn't Seem Open	5	*'I guess showing up is a form of vulnerability but overall I didn't think people felt comfortable or ready to be vulnerable'*
Neutral Comments	3	*'Appropriate boundaries were clearly set by the teacher for self-disclosure and how we responded to each other's comments.'*
Miscellaneous	3	*'I think we were not encouraged to share very private information but I cannot swear to that being a specific bit of guidance'*

When asked to describe opportunities given for informal interaction, the majority of the 56 comments (*n* = 23) stated that opportunities for informal interaction were embedded within each session. A further 13 commented that connection was facilitated at the end of the course. Some (*n* = 7) stated that nothing was offered, whilst three participants had chosen not to engage with whatever was available. There were a further 13 miscellaneous comments on this subject.

There were 40 comments about group connection. The majority of these (*n* = 14) said that connection had been somewhat lacking online. However, a further 11 indicated that connection was satisfactory given the limitations of being online whilst 11 others observed that their experience had been one of positive connections. There were four miscellaneous responses to this question.

Regarding group safety, relatively few participants responded (*N* = 26). Of these, half (*n* = 13) felt safe to share personal information within the group. Another five participants had not felt safe to share, three made neutral remarks and five comments did not fit in with any particular theme.

The majority of 29 comments regarding group openness (*n* = 18) indicated that they had observed others making, or had themselves made, personal disclosures within the group. A further five participants considered their group to not have been open or felt able to make themselves vulnerable. Additionally, there were three comments that were neutral and three more that were miscellaneous.

### Overall advantages and disadvantages of learning an MBP online

Participants were asked to comment on the advantages and disadvantages of having learned an MBP online. This question generated 240 comments, summarised in Table 8.

**Table 8:** Advantages and Disadvantages of Learning an MBP Online

**Advantages of online**		
*N*: 240	*n*	
Convenience	73	*'It was more convenient. No hassle and time spent getting to the venue and back'*
Acessibility	55	*'I would not have been able to attend otherwise as not available near me.'*
Physical Comfort/Safety	33	*'It was more physically comfortable. (especially good to avoid public spaces and public transport at the height of the pandemic).'*
Group Diversity	25	*'There were people from other countries which was great. Different time zones and at the same time we all have very similar challenges.'*
Pedagogical Advantages	16	*'I feel online was less distracting than an in-person course might have been. I get the feeling that generally participants listened more and interrupted less'*
Psychological Comfort/Safety	15	*' I can be quiet in group settings but felt completely different online; maybe the safety of being behind a screen.'*
Negative responses	10	*'Online courses are shallow experiences that are fatiguing & lack engagement. I much prefer classes in real-life.'*
Other Advantages	3	*'I was able to challenge my own families ‘anti mindfulness standpoint’ by actively being involved in the sessions in my own home!'*
Miscellaneous	10	
**Disadvantages of Online**		
*N*: 167	*n*	
Lack of Connection	96	*'I didn't feel like I got to know the teacher or other participants very much. It felt somewhat isolated. I didn't share very much personal information'*
No Disadvantage Experienced	22	*'None. I have attended many mindfulness and meditation course in person and experienced no disadvantage in this on line course'*
Home Environment Lacking	11	*'The setting would have made it easier during the meditations not to have patterns of thoughts associated with my room.'*
Technological Disadvantages	11	*'not secure from other distractions especially digital distractions as using a computer'*
Other Disadvantages	9	*'If I took an in-person course, I would likely have taken it locally, so I will be learning with a group of people who speak my mother tongue'*
Pedagogical Disadvantages	8	*'reduced commitment easy to disassociate from the process'*
Home Environment Distracting	7	*'Easy to just click back into everyday life and stresses'*
Miscellaneous	5	

The majority (*n* = 73) referred to the convenience of online learning (for example, travel time and associated challenges that had been avoided). A further 55 participants mentioned accessibility, stating that they would not have been able to attend an in-person course, because of geographical, financial, health, or other barriers. Physical safety, particularly in the context of the pandemic, and general comfort were cited as advantageous by 33 participants. An additional 25 participants commented favorably on their experience of group diversity. Pedagogical advantages were noted by 16 participants, for example a perception that their distractibility was lessened online and that being online was a ‘leveller’, whilst a further 15 responses referred to experiences of enhanced psychological safety.

When asked to consider the disadvantages of having experienced an MBP online, 167 participant comments were received. Lack of interpersonal connection was by far the most common response (*n* = 96), whilst 22 people commented that they had not experienced any disadvantage. The home environment was considered somewhat deficient by 11 participants and a further 11 recognised that they had encountered disadvantages due to technology, for example distractions from the computer. Assorted disadvantages were noted by nine, whilst eight others perceived pedagogical disadvantages, for example reduced commitment and ease of dissociation from the process. A further seven people considered that their home environment had been distracting, and five others made comments that were not classifiable.

## Discussion

Participants’ responses to the survey suggested that their experiences of learning MBPs online was largely very positive. They particularly valued the opportunity to connect with other group members, and learn new skills, and some said that learning mindfulness online had been lifechanging. The few disadvantages were around a perceived loss of connection with fellow participants compared to what they would have expected or had previously experienced from other in-person courses, and a tendency to get distracted in the online environment.

### Who attends online MBPs?

The most frequently occurring demographic traits in online MBP participants were white, middle-aged, and female. These participants were also highly educated (fewer than ten percent were non-graduates) and lived in households that are more affluent than the average British household, which is consistent with the demographic data relating to in-person mindfulness training [34]. People displaying a majority of these characteristics would have anecdotally been typical attendees at in-person mindfulness trainings suggesting that the systemic barriers to mindfulness learning identified [32] may not have significantly changed by a shift to online delivery. However, our research also identified that online communities were often diverse; survey participants were drawn from 29 different countries around the world, and people from a wide age range had attended the programs. A substantial proportion of those attending an online MBP explicitly stated that they had particularly valued the internationalism of their online groups.

The online groups included more young people and more men than the authors perceived might have been the norm at an in-person MBP [33] and many participants reported facing geographic, health, and childcare barriers to attending an in-person program yet were fully able to participate online. Sexuality and ethnicity were the demographic factors that seemed least diverse in the online setting. It is difficult to say how representative this sample of 161 MBP participants is of the population who has attended an online MBP, as there is no data on number of people who have undertaken an MBP.

### What participants think about learning mindfulness online

Given that most participants had voluntarily paid to join an MBP it seems probable that they were inherently satisfied with the concept of the online medium before signing up (or at least willing to take a risk on its satisfactoriness) and motivated by the practical benefits of learning online. This was borne out by the data—experiences of the online setting were considered overwhelmingly positive, though a few people who had before attended an in-person MBP made negative comparisons of the online MBP with their prior experiences of in-person MBPs.

Participants reported that they felt connected with and supported by their teacher. Connection with the teacher is important both in terms of participant feelings of safety and their ability to ‘catch’ embodied mindfulness from their teacher who communicates mindfulness through the process as well as teaching content [30]. Despite reporting feeling connected with the teacher and with the group, the primary disadvantage identified by online participants was the lack of opportunity to connect more fully with fellow attendees. This connection deficit may have been heightened because some participants undertook an MBP during a pandemic lockdown period when in-person courses were unavailable and social contact would likely have been substantially restricted. However given the importance of the group process to the implicit MBP curriculum, this seems worthy of further consideration.

### Pedagogical issues of online learning

Several pedagogical issues were identified. Survey responses showed that many participants felt easily distracted during the teaching sessions–and this appears to be a major challenge when engaging with content digitally. It seems likely that social norms around engagement and courtesy (e.g. checking 'phones whilst in class) may be bypassed far more readily in the online setting and that distractions are likely to be incoming via the electronic device or in the participants’ local environment. Whilst many participants said that they had received guidance around managing distractions prior to joining the program (e.g., finding a quiet and private space) it seems that teaching organisations could consider offering more precise guidance about ways in which distractions could be minimised or worked with, and that it may be beneficial for the field to develop additional guidelines for online MBPs. However, we also acknowledge that removing all distractions is never going to be feasible, nor even desirable, since identifying these and learning to relate to and manage them differently presents an invaluable opportunity to practice mindfulness. Guidance around how to work with inevitable distractions that arise during an online MBP could, for example, be made explicit by MBP teachers and used to aid participant learning about mindfulness.

Despite having only ever met online, participants identified many positive and mindful attitudes in teachers. The most common description of their teacher was ‘warm’. Patience, non-judgment, and acceptance were mentioned explicitly when describing characteristics of their teachers, whilst other adjectives conveyed attitudes of curiosity, trust, generosity, non-striving, gratitude, and beginner’s mind. The words chosen to describe teachers suggest that their embodiment of mindfulness (which is about being connected with supportive attitudinal qualities and present moment bodily sensations) was conveyed sufficiently to have been recognised by the vast majority of those attending the virtual group. This suggests that it is possible to convey a crucial element of the implicit curriculum even when the group is not physically present in the same room.

However, several participants reported that non-verbal cues were not always easy to read. Some participants specifically stated that it was difficult to know when to talk and that this was uncomfortable; best practice might be for teachers to agree processes around turn-taking with their group at the start of the program, for example possible use of ‘digital hands up’. Given that the field has obtained several years’ experience of delivering MBPs online, it may benefit from collectively reviewing these experiences. It would be helpful to identify any areas where online implementation may have been constrained by theory and practice more relevant to in-person teaching, in case further adaptations could be added to work previously undertaken [37] to optimise online delivery and communication of the more subtle aspects of the MBP curriculum.

Some participants said that they were more able to engage online than would have been possible in-person and found that being together in that way was ‘a leveller’, presumably because the digital setting rendered it more difficult to discern information about others’ backgrounds and to make related judgments (e.g. a participant with impaired mobility would have been able to choose whether to disclose what would, in-person, have been an obvious disability to the group). Participants generally appreciated a sense of choice around having cameras on or off, though may not have recognised the impact of their choices on safeguarding and group process. Being in their own home improved feelings of comfort and both physical (avoidance of COVID-19) and psychological safety for some participants. A downside that was identified was the increased likelihood of being interrupted or overheard by fellow householders.

### Clinical implications

Online delivery presented some challenges to participant engagement – distractibility and subsequent multi-tasking during the classes were common occurrences. Specific guidance about handling these appears important given the distractions are not always controllable in the home environment and are inherently likely when using an online device to access MBP training. Being readily distracted by notifications or other applications is something that participants are often advised to manage. Responding to unexpected digital interruptions could be an interesting explicit point made during teaching, given that attention control is a central component of mindfulness training [38, 39]- it seems important to consider and plan for this. Guidelines around how to work with inevitable distractions that arise during an online MBP could be made explicit by MBP teachers and used to aid participant learning about mindfulness.

Pre-course orientation was not always as comprehensive as Good Practice Guidelines [6] recommend, with 28% reporting they received no orientation at all. The responses to this question suggest that further study of online MBP implementation may be worthwhile. At present there is no data to compare this to in-person MBP courses, so it is unknown how widespread lack of course orientation is in the field, or if it is more likely in an online setting, or perhaps necessarily communicated differently and not identified as orientation by the participant. This is an important finding since pre-course orientation is essential for both teacher and participant to gain assurance that it is the right time for the participant to undertake the program. Initial conversations of this type enable the program provider to assess suitability to safeguard potential participants from risks that could be heightened, if for example, they were experiencing severe mental health challenges or a difficult life experience at the time when they would be encouraged to focus on their inner experiences and turn towards difficulties.

The extent to which group process was facilitated also seemed to be variable. Some participants experienced multiple different vehicles for building rapport and connection with their virtual group whilst others expressed regret at not having been afforded many, or even any, such opportunities by their MBP teacher. Given that participants frequently reported a lack of ‘groupness’ (as did telehealth participants [14]), it might be of benefit to the field to document guidelines as to minimum expectations around facilitating group interaction. The feeling of the group seems to be a vital component of the implicit curriculum and so providing training and guidance for teachers in how best to foster group connectivity among participants online seems worth prioritising.

### Future research

It would be useful for further research to be undertaken regarding pedagogical aspects of online mindfulness learning, such as how teachers can support participants to manage distractions, and indeed use these explicitly as a source of learning about mindfulness and how informal interpersonal connections between participants might be more overtly cultivated online. These themes are likely to be generalisable to other online learning scenarios beyond the mindfulness field. The home environment was identified as positive in terms of convenience, comfort, feelings of safety, and integration of mindfulness practice into everyday life, but some participants found that environment distracting and said that going elsewhere to learn mindfulness would have been supportive. It would be helpful to understand more about how being online at home (or in the workplace) can support or impede learning and cultivation of mindfulness practice. Since online delivery of MBPs is here to stay and was well-received by these participants, it seems important for future research to explore what might further enhance the experience for participants and to optimise their experiences of the implicit curriculum. These considerations would also support the broadening of access to populations who would benefit from potentially transformational learning but are not yet being reached.

### Limitations

It is possible that the delay between completing the course and responding to the survey may mean that positive or negative experiences of learning online could have been exaggerated or forgotten. We did not ask survey respondents whether they undertook their MBP during a lockdown period, which may have influenced their answers (for example views about insufficient connection with others may reflect them having been amidst a global pandemic rather than being specific to MBPs delivered online). Participants’ perspectives may also have been somewhat influenced by previous experiences of mindfulness. It was clear from their responses that some participants had previously undertaken mindfulness courses, both in-person and online, and that some were training to teach mindfulness and therefore more experienced practitioners—we did not specifically ask about this and therefore did not gather potentially useful contextual information.

Another limitation is there was no data collected on participants’ mental health status or neurodivergence, this may have added relevant information regarding the people who are motivated to join online MBPs as well as perhaps highlighting themes regarding any specific benefits or difficulties they reported. Similarly, we did not specifically ask any questions relating to participants’ experiences of difficulty on the course, although there was scope for this to have been provided in response to open questions.

## Conclusions

Overall, participants found learning MBPs online extremely beneficial and highly convenient, and they valued the international nature of their groups. Participants also reported that they were readily distracted in the online environment, and many identified a sense of loss regarding relational connection with other participants. The findings of this study suggest that although MBPs are effective and highly rated by participants who engage with them digitally, there are opportunities for both individual teachers and the wider field to consider adjustments that would further enhance online participants’ experiences. These could include offering mindfulness teachers more specific guidance – having gained years of online experience it might be timely to review the MBI:TAC addenda [37]). Additionally, it seems sensible for teacher training curricula to highlight factors that are specific to the online environment, for example to include practices that incorporate working with digital distraction. Mindfulness supervisors could also be prompted to focus on best practice online (e.g. strengthening group process, managing camera use), although in reality it is likely that these aspects would be covered by current supervision practice. We identified that orientation processes for online mindfulness courses were somewhat variable and so perhaps the field might document what ought to be covered in orientation. This summary could include provision for concerns such as distractibility, use of cameras and participation in the group which should be communicated before the course begins, to enhance the experience for all concerned. Working to optimise the offerings should be a high priority for all who are interested in the mindfulness field since online MBPs have significant potential to benefit both the individual and the wider societies they operate in.

## Data Availability

The original dataset analysed during the study is not publicly available, to protect participant privacy.

## Abbreviations

MBSR: Mindfulness-Based Stress Reduction
MBP: Mindfulness-Based Program
BAMBA: British Association of Mindfulness-Based Approaches
MBCT: Mindfulness-Based Cognitive Therapy
USA: United States of America
UK: United Kingdom
SPSS: Statistical Package for the Social Sciences
GBP: Great British Pounds
